# Fermentative production and direct extraction of (−)-α-bisabolol in metabolically engineered *Escherichia coli*

**DOI:** 10.1186/s12934-016-0588-2

**Published:** 2016-11-08

**Authors:** Gui Hwan Han, Seong Keun Kim, Paul Kyung-Seok Yoon, Younghwan Kang, Byoung Su Kim, Yaoyao Fu, Bong Hyun Sung, Heung Chae Jung, Dae-Hee Lee, Seon-Won Kim, Seung-Goo Lee

**Affiliations:** 1 Synthetic Biology and Bioengineering Research Center, Korea Research Institute of Bioscience and Biotechnology (KRIBB), Daejeon, 34141 Republic of Korea; 2 Biosystems and Bioengineering Program, University of Science and Technology (UST), Daejeon, 34113 Republic of Korea; 3 Department of Chemical and Biological Engineering, Korea University, Seoul, 02841 Republic of Korea; 4 Department of Biotechnology, Chonnam National University, Yeosu, 550749 Republic of Korea; 5 Bioenergy and Biochemical Research Center, Korea Research Institute of Bioscience and Biotechnology (KRIBB), Daejeon, 34141 Republic of Korea; 6 Division of Applied Life Science (BK21 Plus), PMBBRC, Gyeongsang National University, Jinju, 52828 Republic of Korea

**Keywords:** (−)-α-Bisabolol, (−)-α-Bisabolol synthase, Mevalonate pathway, Farnesyl diphosphate synthase, In situ extraction, Vegetable oils, *Escherichia coli*

## Abstract

**Background:**

(−)-α-Bisabolol, also known as levomenol, is an unsaturated sesquiterpene alcohol that has mainly been used in pharmaceutical and cosmetic products due to its anti-inflammatory and skin-soothing properties. (−)-α-Bisabolol is currently manufactured mainly by steam-distillation of the essential oils extracted from the Brazilian candeia tree that is under threat because its natural habitat is constantly shrinking. Therefore, microbial production of (−)-α-bisabolol plays a key role in the development of its sustainable production from renewable feedstock.

**Results:**

Here, we created an *Escherichia coli* strain producing (−)-α-bisabolol at high titer and developed an in situ extraction method of (−)-α-bisabolol, using natural vegetable oils. We expressed a recently identified (−)-α-bisabolol synthase isolated from German chamomile (*Matricaria recutita*) (titer: 3 mg/L), converted the acetyl-CoA to mevalonate, using the biosynthetic mevalonate pathway (12.8 mg/L), and overexpressed farnesyl diphosphate synthase to efficiently supply the (−)-α-bisabolol precursor farnesyl diphosphate. Combinatorial expression of the exogenous mevalonate pathway and farnesyl diphosphate synthase enabled a dramatic increase in (−)-α-bisabolol production in the shake flask culture (80 mg/L) and 5 L bioreactor culture (342 mg/L) of engineered *E. coli* harboring (−)-α-bisabolol synthase. Fed-batch fermentation using a 50 L fermenter was conducted after optimizing culture conditions, resulting in efficient (−)-α-bisabolol production with a titer of 9.1 g/L. Moreover, a green, downstream extraction process using vegetable oils was developed for in situ extraction of (−)-α-bisabolol during fermentation and showed high yield recovery (>98%).

**Conclusions:**

The engineered *E. coli* strains and economically viable extraction process developed in this study will serve as promising platforms for further development of microbial production of (−)-α-bisabolol at large scale.

**Electronic supplementary material:**

The online version of this article (doi:10.1186/s12934-016-0588-2) contains supplementary material, which is available to authorized users.

## Background

**Fig. 1:**
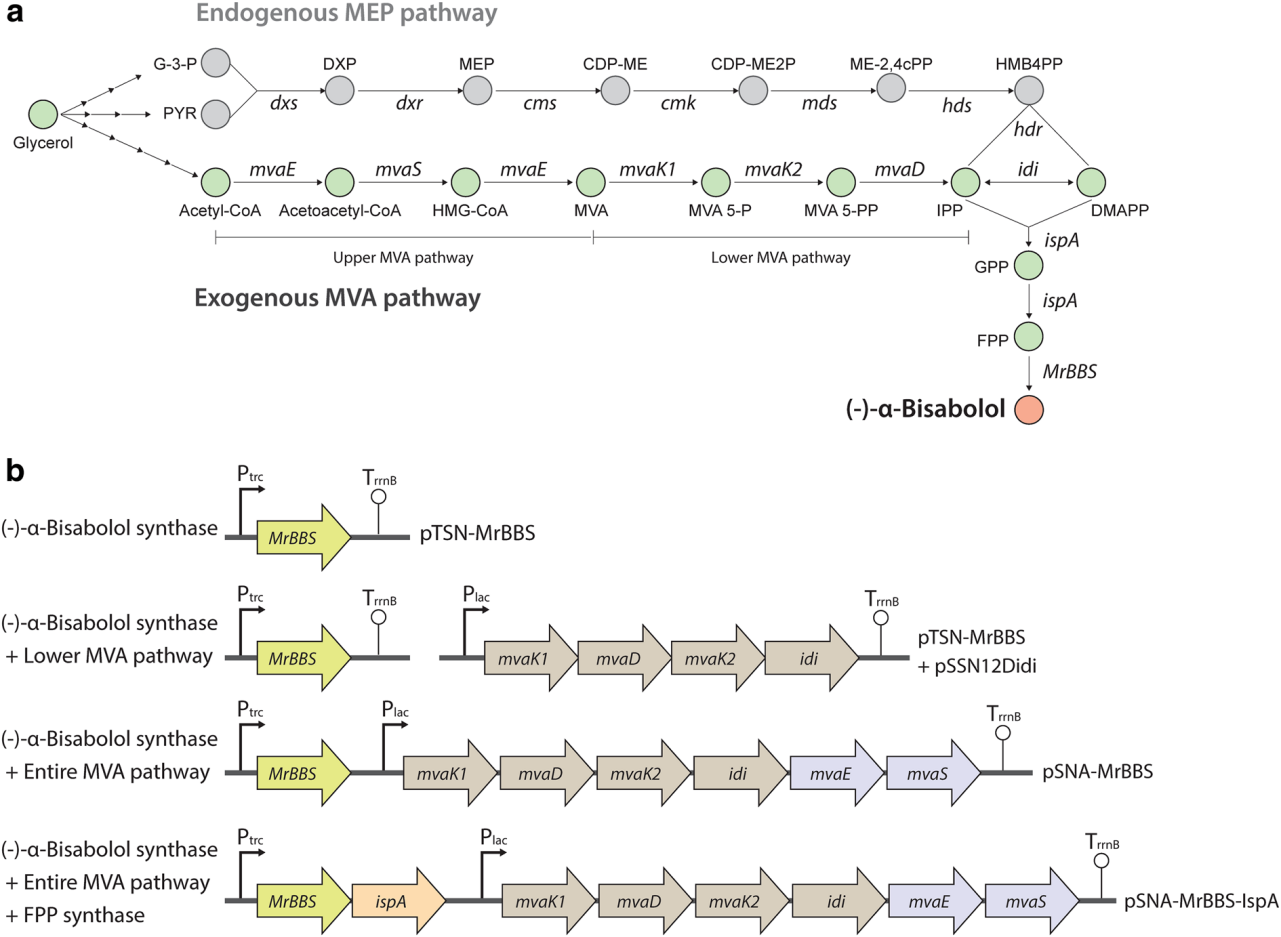
Biosynthesis of (−)-α-bisabolol in engineered *E. coli*. **a** Schematic representation of (−)-α-bisabolol production in an engineered *E. coli* harboring endogenous 2-C-methyl-d-erythritol 4-phosphate (MEP) pathway genes and exogenous mevalonate (MVA) pathway genes. The native MEP pathway consists of: *DXS* deoxyxylulose 5-phosphate synthase; *DXR* deoxyxylulose 5-phosphate reductoisomerase; *CMS* 2-C-methylerythritol 4-phosphate cytidyl transferase; *CMK* 4-(cytidine 5′-diphospho)-2-C-methylerythritol kinase; *MDS* 2-C-methylerythritol 2,4-cyclodiphosphate synthase; *HDS* (E)-4-hydroxy-3-methylbut-2-enyl diphosphate reductase; and *HDR* hydroxymethylbutenyl diphosphate reductase. The engineered mevalonate pathway consists of seven enzymes: MvaE, acetoacetyl-CoA thiolase (GenBank accession number: AF290092); MvaS, 3-hydroxy-3-methylglutaryl- CoA synthase (GenBank accession number: AF290092); MvaA, 3-hydroxy-3-methylglutaryl-CoA reductase (GenBank accession number: AF290092); MvaK1, mevalonate kinase (GenBank accession number: AF290099); MvaK2, phosphomevalonate kinase (GenBank accession number: AF290099); MvaD, mevalonate 5-diphosphate decarboxylase (GenBank accession number: AF290099); Idi, isopentenyl diphosphate isomerase (GenBank accession number: AF119715); and IspA, geranyl diphosphate synthase or farnesyl diphosphate synthase (GenBank accession number: AAC73524). MrBBS; *E. coli* codon-optimized (−)-α-bisabolol synthase of German chamomile (GenBank accession number: KU680479). **b** Plasmid constructs used for (−)-α-bisabolol production in *E. coli*. pTSN-MrBBS, pTrc99A with *MrBBS* gene from German chamomile; pSSN12Didi, pSTV28 containing *mvaK1, mvaD, mvaK2* of *Streptococcus pneumoniae,* and *idi* of *Escherichia coli*; pSNA-MrBBS, pSTV28 containing *mvaK1*, *mvaK2, and mvaD* of *S. pneumoniae, idi* of *E. coli, mvaE* and *mvaS* of *Enterococcus faecalis*, and *MrBBS* of German chamomile; pSNA-MrBBS-IspA, pSNA-MrBBS containing *ispA* of *E. coli*

Terpenoids, also known as isoprenoids, are one of the largest and most diverse classes of naturally occurring products [1]. In animals, terpenoids play crucial roles as membrane constituents (e.g., cholesterol) and components of the respiratory electron transport chain (e.g., ubiquinone) [2], whereas in microbes and plants, they are found as secondary metabolites that have been used as pharmaceuticals (e.g., paclitaxel and artemisinin), flavors, and fragrances (e.g., menthol and patchoulol) [3]. Terpenoids, including monoterpenes, sesquiterpenes, and diterpenes, are synthesized by terpene synthases (TPSs) with universal five-carbon building units, isopentenyl diphosphate (IPP) and its isomer dimethyl allyldiphosphate (DMAPP). IPP and DMAPP are synthesized in two different pathways: the mevalonate (MVA) pathway [4], and the 2-C-methyl-d-erythritol 4-phosphate (MEP) pathway [5] (Fig. 1a). In general, Gram-negative bacteria, including *Escherichia coli*, employ the MEP pathway, whereas eukaryotes, archaea, humans, and Gram-positive cocci use the MVA pathway [6]. Prenyltransferases assemble the IPP and DMAPP units to produce the prenyl diphosphates, which are terpenoid precursors, including geranyl diphosphate (GPP, C_10_), farnesyl diphosphate (FPP, C_15_), and geranyl geranyl diphosphate (GGPP, C_20_). These prenyl diphosphate molecules are converted into a large variety of terpenoids by TPSs [7, 8].

(−)-α-Bisabolol is an unsaturated sesquiterpene alcohol that occurs naturally in the Brazilian candeia tree (*Eremanthus erythropappus*) and in medicinal herbs such as German chamomile (*Matricaria recutita*) [9, 10]. It has been shown to have pharmaceutical functions (e.g., antibacterial, antiseptic, and anti-inflammatory activities), and skin-soothing and -moisturizing properties [11–13]. Owing to the low toxicity associated with (−)-α-bisabolol, the Food and Drug Administration (FDA) has granted it the Generally Regarded as Safe (GRAS) status that has promoted its use as an active ingredient in several commercial products [14]. At present, natural (−)-α-bisabolol is primarily manufactured through the steam-distillation of candeia essential oils extracted from the Brazilian candeia tree, which has raised environmental and bioconservation issues in recent years [10]. (−)-α-Bisabolol can also be chemically synthesized; however, chemical synthesis requires an additional economically unviable purification step because of the formation of other diastereomers ((+)-α-bisabolol and (±)-*epi*-α-bisabolol) and undesirable byproducts [15]. Therefore, a sustainable supply of nature-identical (−)-α-bisabolol is essential to specialty chemical industries. Recently, a (−)-α-bisabolol synthase capable of synthesizing enantio-selective (−)-α-bisabolol as a single terpenoid product has been identified in German chamomile. (−)-α-bisabolol synthase was expressed in *Saccharomyces cerevisiae* for the production of (−)-α-bisabolol and had a yield of 8 mg/L after 4 days of cultivation [15]. This previous study has paved the way for the biological production of enantioselective (−)-α-bisabolol in engineered microorganisms, but the productivity is very low for industrial production of (−)-α-bisabolol.

The aim of this study was to produce (−)-α-bisabolol by large-scale fermentation of *E. coli*, which has been widely used for various industrial applications, and to develop an economically viable direct (−)-α-bisabolol extraction process during fermentation. To achieve this, we created (−)-α-bisabolol-producing *E. coli* in three steps: (1) We introduced the German chamomile (−)-α-bisabolol synthase gene (*MrBBS*) into *E. coli* convert endogenous FPP to (−)-α-bisabolol; (2) We engineered the exogenous MVA pathway to increase IPP and DMAPP pool; and (3) We overexpressed an *ispA* gene encoding an FPP synthase that efficiently provides the (−)-α-bisabolol precursor (FPP) from IPP and DMAPP (Fig. 1b).

## Methods

### Bacterial strains, media, and culture conditions

Strains used in this study are listed in Table 1. *E. coli* DH5α was used for cloning experiments and (−)-α-bisabolol production. BL21(DE3) and MG1655 strains were used to compare effects of different host strains on the production and toxicity of (−)-α-bisabolol. *E. coli* strains were grown in Luria Bertani (LB) medium (10 g/L tryptone, 5 g/L yeast extract, and 5 g/L NaCl) for cloning experiments and (−)-α-bisabolol production. To produce (−)-α-bisabolol in *E. coli*, we used various media at 30 °C and 200 rpm; terrific broth (TB) medium (12 g/L enzymatic casein digest, 24 g/L yeast extract, 9.4 g/L K_2_HPO_4_, and 2.2 g/L KH_2_PO_4_), 2xYT medium (16 g/L tryptone, 10 g/L yeast extract, and 5 g/L NaCl), and M9 minimal medium (6.78 g/L Na_2_HPO_4_, 3 g/L KH_2_PO_4_, 0.5 g/L NaCl, 1 g/L NH_4_Cl, 0.241 g/L MgSO_4_, 0.0111 g/L CaCl_2_, and 0.1 g/L thiamine). M9 minimal medium was supplemented with 4 g/L of glucose. Ampicillin (100 μg/mL), chloramphenicol (34 μg/mL), or isopropyl β-d-1-thiogalactopyranoside (IPTG) as required.

**Table 1:** List of strains, plasmids, and primers used in this study

Name	Description	Refs
Strains		
DH5α	*F* ^−^ *, Φ80lacZ·ΔM15·ƒ(lacZYA*−*argF)U169 deoR recA1 endA1 hsdR17(rk*−*, mk*+*) phoA supE44 thi*-*1 gyrA96 relA1*	Enzynomics
MG1655	F^−^, λ^−^, *ilvG* ^−^, *rfb*-50, *rph*1	ATCC 700926
BL21(DE3)	BL21 F^−^, *dcm ompT hsdS (rB*- *mB*-*) gal* λ(DE3)	Enzynomics
Plasmids		
pSSN12Didi	pSTV28 containing *mvaK1, mvaD, mvaK2* of *Streptococcus pneumoniae,* and *idi* of *E. coli*	[19]
pPROLar.A	P_l*ac/ara*-*1*_ expression vector, Kan^r^, p15A ori	Clontech
pTrc99A	P_*trc*_ expression vector, Amp^r^, lacI^q^, pBR322 ori	GE Healthcare
pTSN-MrBBS	pTrc99A containing *MrBBS* of *Matricaria recutita*	This study
pPR-IspA	pPROLar.A containing *ispA* of *E. coli*	This study
pTSN-MrBBS-IspA	pTSN-MrBBS containing *ispA* of *E. coli*	This study
pSNA	pSTV28 containing *mvaK1*, *mvaK2, and mvaD* of *S. pneumoniae, idi* of *E. coli, and mvaE* and *mvaS,* of *Enterococcus faecalis*	[19]
pSNA-MrBBS	pSNA containing *MrBBS* of *M. recutita*	This study
pSNA-MrBBS-IspA	pSNA-MrBBS containing *ispA* of *E. coli*	This study
Primers^a^		
Bis-IF	aggttaaaccatgagcacactgagcgtcag	This study
Bis-IR	cgactctagattagactatcatcggatgta	This study
Bis-VF	gatagtctaatctagagtcgacctgcaggc	This study
Bis-VR	gtgtgctcatggtttaacctcctgtgtgaaattgttatc	This study
IspAI-F	ggtaccccatatggactttccgcagcaact	This study
IspAI-R	tgcctctagattatttattacgctggatga	This study
IspAV-F	taataaataatctagaggcatcaaataaaa	This study
IspAV-R	gaaagtccatatggggtacctttctcctct	This study
IspAop-IF	acatccgatgatagtctaatattcattaaagaggagaaag	This study
IspAop-IR	tgcatgcctgcaggtcgactctagattatttattacgctg	This study

Overlap region for Gibson Assembly is underlined

^a^Primer sequences are indicated in the 5′–3′ direction

*n*-Dodecane was used as an overlay to prevent the loss of volatiles. Furthermore, *n*-dodecane was used to solubilize and extract (−)-α-bisabolol, which is toxic to *E. coli* cell growth in high concentrations, from the culture media during cultivation. MVA was prepared from mevalonolactone (Sigma Aldrich) as previously described [16]. Cell growth was monitored by measuring the optical density at a wavelength of 600 nm (OD_600_) with a spectrophotometer (Ultrospec 8000, GE Healthcare, Uppsala, Sweden). The inhibitory effect of (−)-α-bisabolol on the growth of *E. coli* strains was investigated in LB liquid medium supplemented with various concentrations of (−)-α-bisabolol. Diluted samples from cultures during the stationary phase were plated on LB solid medium and the colony-forming units (CFUs) were determined after overnight incubation at 30 °C.

### Plasmid construction

Plasmids and polymerase chain reaction (PCR) primers used in this study are listed in Table 1. Common procedures, including genomic DNA preparation, restriction digestions, transformations, and other standard molecular biological techniques were performed as previously described [17]. All restriction enzymes and T4 DNA ligase were purchased from New England Biolabs (Ipswich, USA). PCR was performed following the manufacturer’s instructions, using a high fidelity KOD-Plus-Neo polymerase (Toyobo, Osaka, Japan). Plasmid preparation and gel extraction kits were obtained from Promega (Madison, USA) and oligonucleotides were synthesized by Bioneer (Daejeon, Korea). The *MrBBS* gene (GenBank accession number: KJ020282) was optimized to *E. coli* codons and synthesized by Bioneer (Additional file 1: Figure S1). We deposited the nucleotide sequence data of *E. coli* codon-optimized *MrBBS* in the GenBank database (accession number: KU680479). The synthesized *MrBBS* was amplified by PCR with Bis-IF and Bis-IR primers (Table 1) and the plasmid backbone was amplified with the Bis-VF and Bis-VR primers from pTrc99A (GE Healthcare). The two PCR-amplicons were assembled via the Gibson Assembly Method [18] using Gibson Assembly Master Mix (New England Biolabs), to produce the pTSN-MrBBS plasmid. The entire polycistronic MVA pathway genes were inserted into pTSN-MrBBS through *Xba*I restriction site digestion of both the pTSN-MrBBS and the pSNA plasmids containing all the mevalonate pathway genes [19], followed by ligation with T4 DNA ligase. The resultant plasmid was designated pSNA-MrBBS. To overexpress FPP synthase, the *ispA* gene (GenBank accession number: AAC73524) was PCR-amplified from *E. coli* MG1655 genomic DNA, using IspAI-F and IspAI-R primers. To avoid proofreading errors introduced during PCR, the amplified *ispA* gene was first inserted into the *Xba*I restriction site of pTSN-MrBBS (instead of pSNA-MrBBS). The pTSN-MrBBS-IspA and pSNA plasmids were digested with *Xba*I followed by ligation with T4 DNA ligase to create pSNA-MrBBS-IspA. The direction of the MVA pathway genes was verified by Sanger sequencing.

### Batch and fed-batch fermentation

To prepare the pre-culture, the engineered *E. coli* was cultured in 100 mL of TB medium at 30 °C and 180 rpm. The batch fermentation was performed by inoculating 1% (v/v) of pre-culture into 400 mL TB medium supplemented with 10 g/L glycerol and overlaid with 80 mL *n-*dodecane in a 1 L fermenter. The cultures were incubated at 30 °C and air-aerated with a flow rate of 1 volume of air per volume of medium per min (vvm), the agitation speed remained fixed at 200 rpm in all experiments. Glycerol was used as a carbon source at a concentration of 10 g/L. The pH was balanced to pH 7.0 by the addition of 1 M HCl and 25% NH_4_OH solutions. *n*-Dodecane was overlaid up to 20% (v/v) of culture volume to continuously extract (−)-α-bisabolol from the culture broth during fermentation. The fed-batch fermentation began as a batch operation and was switched to the fed-batch mode at 24 h, when OD_600_ reached 3.0. At the beginning of fed-batch fermentation, TB medium was supplemented with 10 g/L glycerol, 0.1% (v/v) trace element solution (27 g/L FeCl_3_·6H_2_O, 2 g/L ZnCl_2_·4H_2_O, 2 g/L CoCl_2_·6H_2_O, 2 g/L Na_2_MoO_4_·2H_2_O, 1 g/L CaCl_2_·2H_2_O, 1.3 g/L CuCl_2_·6H_2_O, and 0.5 g/L H_3_BO_3_) and 1% (v/v) vitamin solution (Sigma Aldrich, Cat. No. M6895). Glycerol was then fed at a rate of 0.01 L/h and 10% (v/v) canola oil (instead of 20% (v/v) *n-*dodecane) was used to overlay 30 L TB medium in a 50 L fermenter.

### (−)-α-Bisabolol extraction with *n-*dodecane and vegetable oils

Extraction of (−)-α-bisabolol was performed using four common vegetable oils: canola, olive, corn, and soybean oil. A total of 0.1 g of (−)-α-bisabolol was added to 10 mL of sterile TB medium in a glass vial and 1 mL of individual vegetable oil was overlaid to the solution. After gentle stirring of the mixture, it was incubated at 25 °C for 1 h to separate the water-based TB medium and oil phase. The aqueous layer was removed and the organic layer was transferred to a new vial for gas chromatography (GC) analysis of (−)-α-bisabolol. The recovery yield of each vegetable oil was compared with that of *n-*dodecane. To determine the recovery efficiency of *n-*dodecane from cell pellets and TB medium, *E. coli* DH5α cells transformed with the pSNA-MrBBS-IspA plasmid were cultivated in TB medium in the presence of 20% (v/v) *n-*dodecane at 30 °C at 180 rpm for 48 h. After centrifugation of the culture at 14,000 rpm for 10 min, the pellet, supernatant, and organic layers of *n-*dodecane were fractionated. To determine the recovery yield of *n-*dodecane, the organic layer was directly subjected to GC analysis of (−)-α-bisabolol. The isolated supernatant was mixed with 20% (v/v) *n-*dodecane, incubated for 30 min, and centrifuged at 14,000 rpm for 10 min. The organic layer was isolated and used for GC analysis of (−)-α-bisabolol. The cell pellet was first disrupted by sonication and the lysate was used for the extraction and quantification of (−)-α-bisabolol through the process described above.

### (−)-α-Bisabolol identification and quantification

To analyze (−)-α-bisabolol production, the culture broth was centrifuged at 13,000 rpm for 10 min to separate the overlaid *n-*dodecane phase. The recovered *n-*dodecane-phase was subsequently analyzed for (−)-α-bisabolol by GC and GC-mass spectrometry (GC–MS). The identification of (−)-α-bisabolol was conducted by GC–MS (5977A MSD) with a HP-5MS column (30 m × 0.250 mm × 0.25 µm, Agilent, Santa Clara, USA). The column flow was maintained at 1 mL/min. The oven temperature was initially held at 60 °C for 2 min, increased by 5 °C/min to 300 °C, and held at 300 °C for 10 min. (−)-α-Bisabolol (Cat. No. 23089-26-1, Sigma Aldrich) was used as the standard, and was confirmed through the GC-MS software, Mass Hunter (Agilent, Santa Clara, USA). The quantification of (−)-α-bisabolol was performed using GC equipped with a flame ionization detector (FID) with a HP-5 column (30 m × 0.320 mm × 0.25 µm) at a flow rate of 1 mL/min. The starting temperature of the oven was 60 °C for 2 min, increased at 5 °C/min to 200 °C, held at 200 °C for 2 min, increased by 50 °C/min to 300 °C, and held at 300 °C for 5 min. Helium was used as the carrier gas with an inlet pressure of 5.58 psi. The (−)-α-bisabolol concentration produced by engineered *E. coli* was determined as follows:

$$(-) {\text{-}} \alpha {\text{-bisabolol }}\left[{\text{mg}}/{\rm L} \right] = \frac{{\left[ {\left( - \right) {\text{-}} \alpha {\text{-bisabolol in }}n-{\text{ dodecane phase}}} \right] \times \left[ {{\text{volume of }}n-{\text{ dodecane phase}}} \right]}}{{\left[ {\text{volume of medium phase}} \right]}}$$

### Analysis of glycerol consumption and byproduct formation

The culture broth was centrifuged and the supernatant was used to quantify glycerol consumption and byproduct formation. Glycerol, acetate, and mevalonate concentrations were determined by high performance liquid chromatography (HPLC, Shimadzu, Kyoto, Japan) with a refractive index detector at 454 nm using Aminex HPX-87H column (1300 mm × 7.8 mm, Bio-Rad, Hercules, USA). Sulfuric acid (0.4 mM) was used as the mobile phase at a flow rate of 0.3 mL/min at 50 °C.

## Results and discussion

### Suitability of *E. coli* DH5α as a host for (−)-α-bisabolol production

**Fig. 2:**
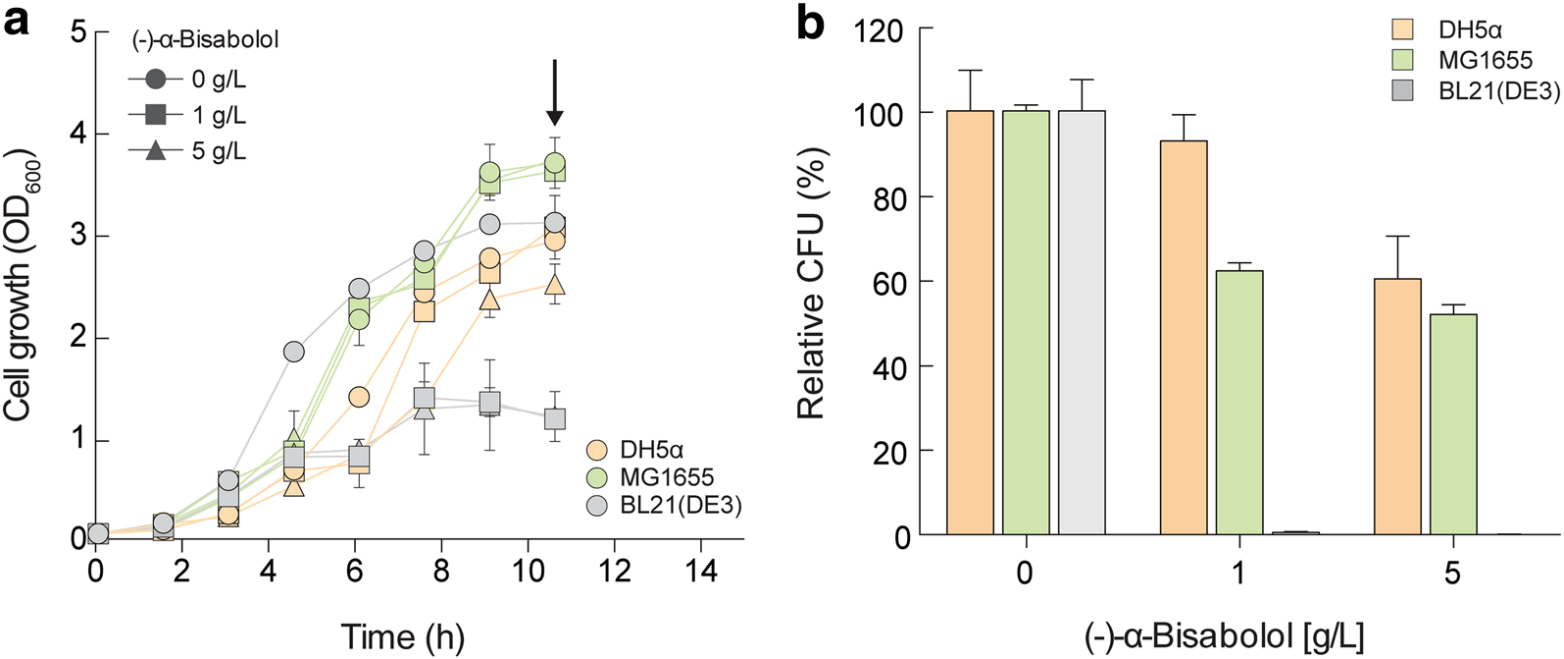
Growth inhibition of *E. coli* strains by exogenous (−)-α-bisabolol. **a** Time course of growth profile. **b** CFU measurement. Cells were grown in LB liquid medium and different amounts of (−)-α-bisabolol were added at the beginning of cultivation. Samples were taken for measurement of optical density at 600 nm (OD_600_). Samples for CFU measurement were taken at early stationary phase (indicated by an *arrow*). Mean values of two independent experiments are given for the CFU measurement

(−)-α-Bisabolol is a naturally occurring sesquiterpene alcohol that exhibits antibacterial activity [13]. This property would be a hurdle for the use of *E. coli* as a terpenoid production host [20]. Therefore, we examined the suitability of three different *E. coli* strains [DH5α, MG1655, and BL21(DE3)] for (−)-α-bisabolol production by conducting growth assays in LB in the presence of various concentrations of (−)-α-bisabolol (Fig. 2). After 3 h of cultivation (mid-log phase), the growth of BL21(DE3) significantly decreased by 1 and 5 g/L (−)-α-bisabolol (Fig. 2a). The growth of MG1655 was not affected by (−)-α-bisabolol at concentrations of 1 or 5 g/L (Fig. 2a). The growth of DH5α slightly decreased at (−)-α-bisabolol concentration of 5 g/L, but growth was not affected by 1 g/L (−)-α-bisabolol (Fig. 2a). This result was corroborated with CFU measurements for each culture. As expected, DH5α showed the best tolerance to (−)-α-bisabolol whereas BL21(DE3) formed no colonies on the solid LB medium (Fig. 2b). These results are consistent with a previous study showing that of five different B- and K-type *E. coli* strains (MG1655, DH5α, S17-1, XL1-Blue, and BL21), DH5α is the best producer of the β-carotene (C40) terpenoid [21]. In addition, *E. coli* DH5α has been used for production of retinol [22], farnesol [23], and protoilludene [23]. We concluded that *E. coli* DH5α is suitable as a host for (−)-α-bisabolol production and it was used for all subsequent experiments.

### (−)-α-Bisabolol production in *E. coli* DH5α expressing the *MrBBS* gene

**Fig. 3:**
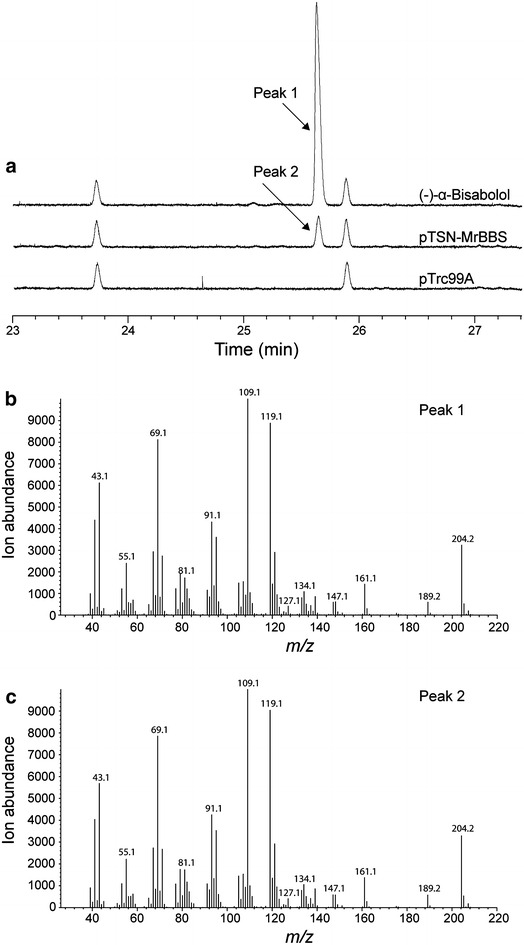
GC and GC–MS analysis of *n*-dodecane extracts from *E. coli* expressing the *MrBBS* gene. **a** Total ion chromatograms of an authentic (−)-α-bisabolol standard (100 mg/L) and the *n-*dodecane extracts from the *E. coli* expressing MrBBS (pTSN-MrBBS) or containing an empty vector (pTrc99A). **b** Mass spectra of (−)-α-bisabolol standard. **c** Mass spectra of (−)-α-bisabolol produced in *E. coli* cells harboring the plasmid pTSN-MrBBS. Each cell was cultivated in LB medium overlaid with 20% (v/v) *n*-dodecane at 30 °C for 48 h

To implement the *de novo* production of (−)-α-bisabolol in *E. coli* DH5α, the gene of *MrBBS*, encoding the (−)-α-bisabolol synthase, was codon-optimized for *E. coli* and introduced via the pTSN-MrBBS plasmid (Fig. 1b). The resulting transformant cells were cultured in LB medium overlaid with 20% (v/v) *n-*dodecane for 48 h. Two-phase cultures have been successfully used to extract toxic, water-immiscible or volatile products [24–26] and *n-*dodecane was successfully used for extraction of (−)-α-bisabolol from the culture broth of *S. cerevisiae* [15]. As expected, in the present study, (−)-α-bisabolol was extracted into the *n-*dodecane phase in the two-phase *E. coli* culture. As the (−)-α-bisabolol was recovered in *n-*dodecane without significant residual amounts left in the cells (Additional file 1: Figure S2), (−)-α-bisabolol purification in a later step will be facilitated. The *n-*dodecane phase was used for GC analysis to measure (−)-α-bisabolol concentrations. There was a peak at 25.6 min in the *n-*dodecane phase sample of *E. coli* DH5α with pTSN-MrBBS, corresponding to the standard (−)-α-bisabolol compound dissolved in *n-*dodecane (Fig. 3a). Mass spectrometry confirmed that the peak at 25.6 min was (−)-α-bisabolol (Fig. 3b, c) and a maximum concentration of 3 mg/L (−)-α-bisabolol was produced by the endogenous MEP pathway and exogenous MrBBS enzyme in *E. coli* DH5α. The peak was not observed in two-phase cultures of DH5α cells containing an empty plasmid as a control experiment (Fig. 3a). The (−)-α-bisabolol synthase gene, *MrBBS*, used in this study was recently isolated from German chamomile and, unlike other TSPs, exclusively synthesizes (−)-α-bisabolol as a single terpene product [15]. Several TPSs producing α-bisabolol as a single major product have been previously cloned and biochemically characterized. However, these enzymes usually produce undesirable isomers with different structures or α-bisabolol of unknown stereochemistry [27–29]. The MrBBS enzyme has shown unique catalytic features including the formation of a single enantiopure (−)-α-bisabolol indicating the possibility of a biotechnological application to natural (−)-α-bisabolol production in *S. cerevisiae* [15]. Here, we have expressed the codon-optimized synthetic *MrBBS* in *E. coli* and successfully produced (−)-α-bisabolol (titer: 3 mg/L). A similar result has been observed when attempting to express other plant terpene synthases in *E. coli* [30, 31].

### (−)-α-Bisabolol production in *E. coli* DH5α expressing *MrBBS* and MVA pathway genes

**Fig. 4:**
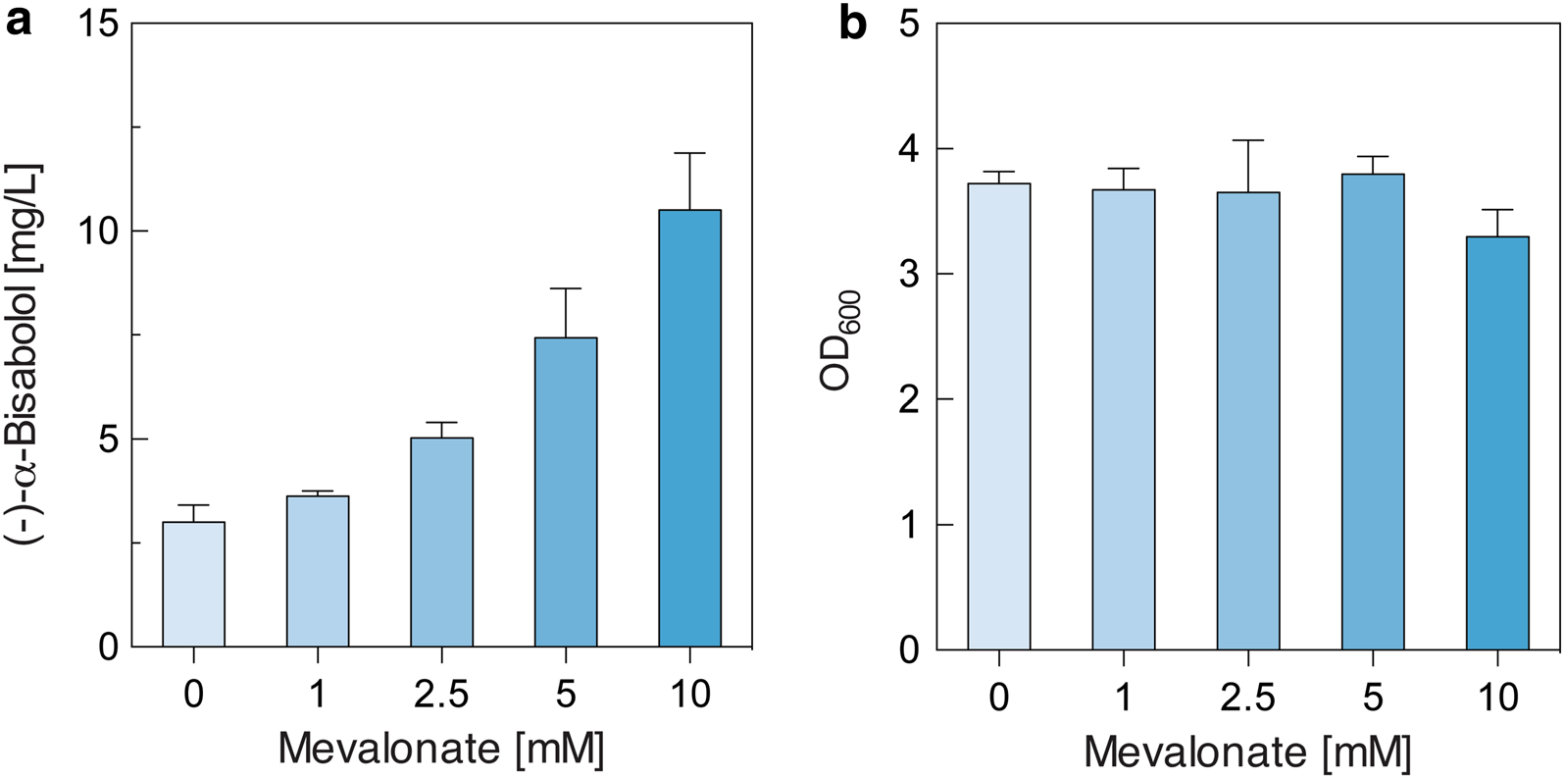
The effect of mevalonate concentration on (−)-α-bisabolol production (**a**) and final OD_600_ (**b**) from engineered *E. coli* harboring pTSN-MrBBS and pSSN12Didi. Cells were grown at 30 °C in LB medium supplemented with different concentrations of mevalonate and 20% (v/v) *n*-dodecane for 48 h. Data represent averages from three replicate cultures;* error bars* show SD

**Fig. 5:**
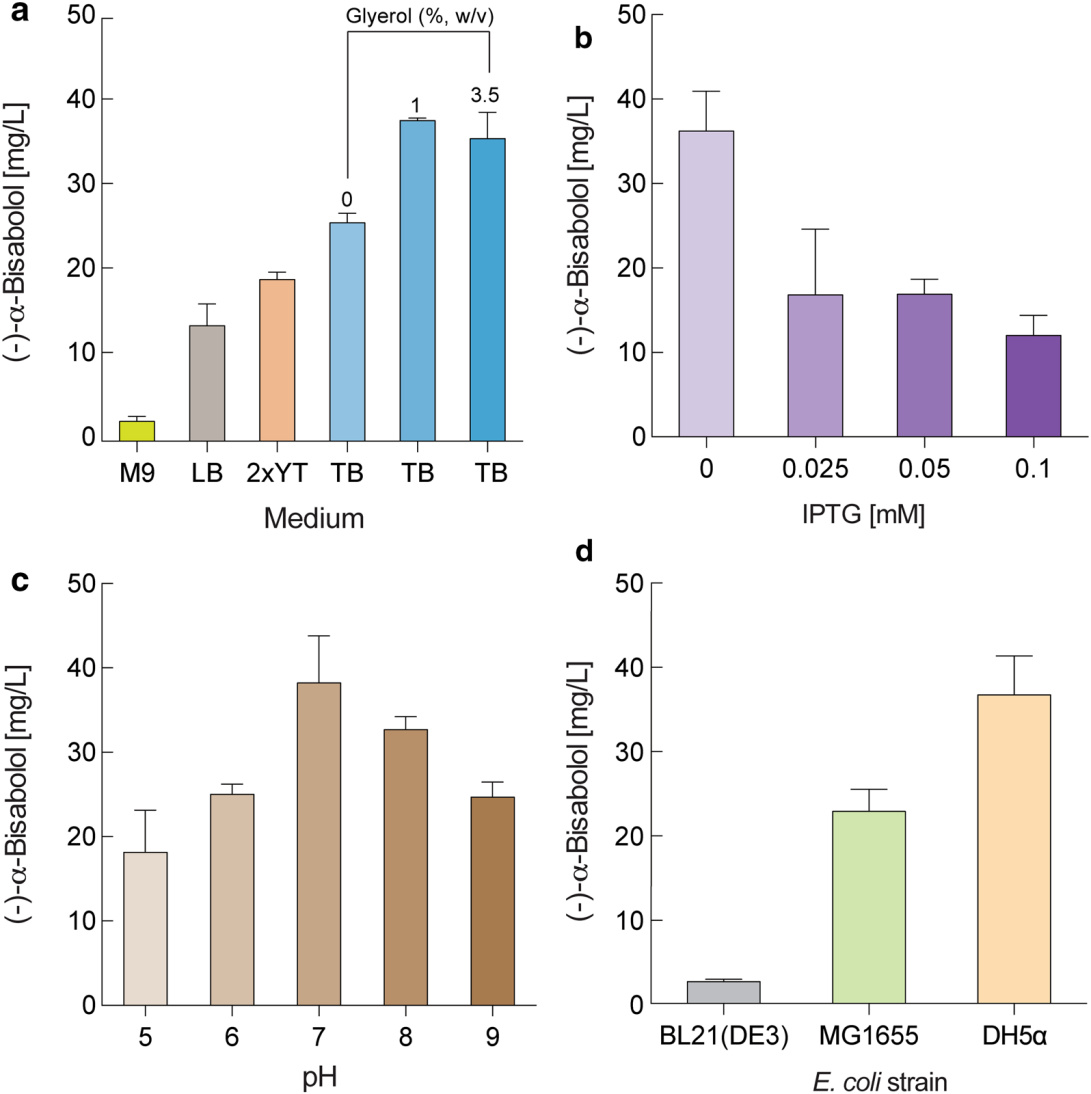
Optimal conditions for (−)-α-bisabolol production in *E. coli* expressing *MrBBS* and all MVA pathway genes. *E. coli* cells harboring pSNA-MrBBS were grown for 48 h at 30 °C in the presence of 20% (v/v) *n*-dodecane with the following variations; **a** Medium (M9 supplemented with 4 g/L glucose, LB, 2xYT, TB) and glycerol concentration; **b** IPTG concentration. When OD_600_ reached 0.5, different concentrations of IPTG were added to the *E. coli* cells grown on TB medium supplemented with 10 g/L glycerol; **c** pH. Different initial pHs were tested to find an optimal pH for (−)-α-bisabolol production. *E. coli* cells were cultivated in TB medium containing 10 g/L glycerol in the absence of IPTG; **d** *E. coli* strains. *E. coli* cells were grown on TB medium, pH 7.0 with 10 g/L glycerol and 20% (v/v) *n*-dodecane in the absence of IPTG. Data represent averages from three replicate cultures and *error bars* show SD

Terpenoid production in microbes is mainly limited by the flux from intermediates of central metabolism (acetyl-CoA for MVA pathway or pyruvate and glyceraldehyde-3-phosphate for MEP pathway) to the substrates of TPSs (GPP, FPP, or GGPP) [32]. The introduction and engineering of the biosynthetic MVA pathway in *E. coli* have improved the production of terpenoids such as amorphadiene [32], carotenoids [19, 21], coenzyme Q10 [33], and farnesol [30] by efficiently supplying IPP and DMAPP. Based on these studies, we exploited the biosynthetic MVA pathway for the biosynthesis of (−)-α-bisabolol in *E. coli*. To increase carbon flux towards TPS substrates, a plasmid (pSSN12Didi) containing the lower MVA pathway genes (Fig. 1b), leading from MVA to IPP and DMAPP [30], was introduced into *E. coli* DH5α expressing the *MrBBS*. MVA is an exogenous substrate for *E. coli*, so supplemented MVA can only be converted to IPP and DMAPP by the engineered MVA lower pathway in *E. coli*. Different concentrations of MVA, ranging from 0 to 10 mM, were examined to determine whether MVA availability limits (−)-α-bisabolol production in LB medium. (−)-α-bisabolol production increased as the concentration of the supplied MVA increased (Fig. 4a) and cell growth was not significantly inhibited (Fig. 4b). With the addition of 10 mM MVA, (−)-α-bisabolol production was 10.5 mg/L, approximately 3.5-fold higher than that obtained without the addition of MVA (3 mg/L, Fig. 4a). Expression of the lower MVA pathway genes in *E. coli* improved (−)-α-bisabolol production with the addition of MVA. However, MVA is not an economically viable substrate. To achieve (−)-α-bisabolol production from cost-effective renewable resources such as glycerol, a major byproduct of the biodiesel industry, we constructed a plasmid pSNA-MrBBS (Fig. 1b). pSNA-MrBBS harbors the complete biosynthetic MVA pathway genes, including the *MrBBS* gene, and enables production of IPP and DMAPP in the absence of MVA. A total of 12.8 mg/L of (−)-α-bisabolol was produced from glycerol in *E. coli* DH5α with pSNA-MrBBS grown in LB (Fig. 5a). This is a slightly higher concentration than that produced in DH5α containing only *MrBBS* and lower MVA pathway genes with the addition of 10 mM MVA (10.5 mg/L). Overall, the expression of biosynthetic MVA pathway genes significantly improved (−)-α-bisabolol production in *E. coli*.

### Optimization of (−)-α-bisabolol production in *E. coli* DH5α expressing *MrBBS* and entire MVA pathway genes

IPP and DMAPP, precursors of FPP, are essential metabolites in *E. coli* that are used for tRNA prenylation, synthesis of quinone and dolichol for respiration, and cell wall biosynthesis [31]. Thus, the metabolic balance of IPP and DMAPP between endogenous essential metabolism and exogenous (−)-α-bisabolol production plays a crucial role in improving production of (−)-α-bisabolol and alleviation of growth inhibition. To determine the optimal IPP and DMAPP pool balancing conditions in the engineered *E. coli* harboring the entire MVA pathway and *MrBBS* genes (pSNA-MrBBS), we examined if cultivation media and glycerol availability limit (−)-α-bisabolol production. To do this we tested different media and concentrations of glycerol ranging from 0 to 3.5% (w/v) (Fig. 5a). *E. coli* DH5α containing the pSNA-MrBBS produced the highest concentration of (−)-α-bisabolol (24.2 mg/L) in TB medium without the addition of an additional carbon source (Fig. 5a). (−)-α-Bisabolol production was dependent on the amount of provided glycerol in the TB medium. With 1% (w/v) glycerol, (−)-α-bisabolol concentration was 38.3 mg/L, approximately 1.4-fold higher than the concentration of (−)-α-bisabolol obtained without the inclusion of glycerol. However, 3.5% (w/v) of glycerol concentration did not show further improvement of (−)-α-bisabolol production (Fig. 5a). Because cell growth of *E. coli* DH5α varied in different culture media, the differences in (−)-α-bisabolol production are partly due to differences in growth (Figure S3A).

Plasmid pSNA-MrBBS contained IPTG-inducible *lac* and *trc* promoters for expression of the complete MVA pathway genes and *MrBBS* gene, respectively (Fig. 1b). To ascertain the optimal inducer concentration for (−)-α-bisabolol production in *E. coli* DH5α with pSNA-MrBBS, induction was conducted with varying concentrations of IPTG ranging from 0 to 0.1 mM. Interestingly, leaky expression of the *MrBBS* and MVA pathway genes in the absence of IPTG showed the highest production of (−)-α-bisabolol (38.9 mg/L). At all other IPTG concentrations (−)-α-bisabolol production decreased as the IPTG concentration increased (Fig. 5b) whereas the growth of *E. coli* DH5α cells is similar regardless of IPTG concentration (Additional file 1: Figure S3B). This result is compatible with a previous report [31] that showed lycopene production in *E. coli* expressing lower MVA pathway genes regulated by the *trc* promoter decreased under all IPTG-induced conditions. In addition, constitutive promoters controlling the expression of MVA pathway genes showed much lower production of amorphadiene than that from IPTG-inducible *lacUV5* and *trc* promoters [34]. High expression of the MVA pathway and *MrBBS* genes under IPTG induced conditions could cause a deficiency of FPP for essential cellular metabolism. Furthermore, the accumulation of toxic intermediates of the MVA pathway, including IPP and 3-hydroxy-3-methyl-glutaryl-CoA (HMG-CoA) [32, 35], could cause growth inhibition along with a decrease of (−)-α-bisabolol production. In addition, we investigated the effects of initial pH of the cultivation of *E. coli* DH5α with pSNA-MrBBS on the production of (−)-α-bisabolol. The optimal pH for (−)-α-bisabolol production was pH 7 (38.2 mg/L, Fig. 5c). To determine the ideal *E. coli* strains for (−)-α-bisabolol production under the optimized culture conditions, we individually introduced the plasmid pSNA-MrBBS into three different *E. coli* strains (DH5α, MG1655, or BL21(DE3)) and measured the (−)-α-bisabolol concentration produced from each. *E. coli* DH5α cells produced the highest amount of (−)-α-bisabolol, and BL21(DE3) showed an approximate 13-fold decrease in (−)-α-bisabolol production compared to DH5α (38.4 mg/L, Fig. 5d). We also measured cell growth during examination of pH effects on (−)-α-bisabolol production in *E. coli* DH5α expressing *MrBBS* and entire MVA pathway genes. Although cell growth slightly decreased at a pH 5 or 6, it was similar at all other pHs (Additional file 1: Figure S3C).

### Effect of the overexpression of FPP synthase on (−)-α-bisabolol production

**Fig. 6:**
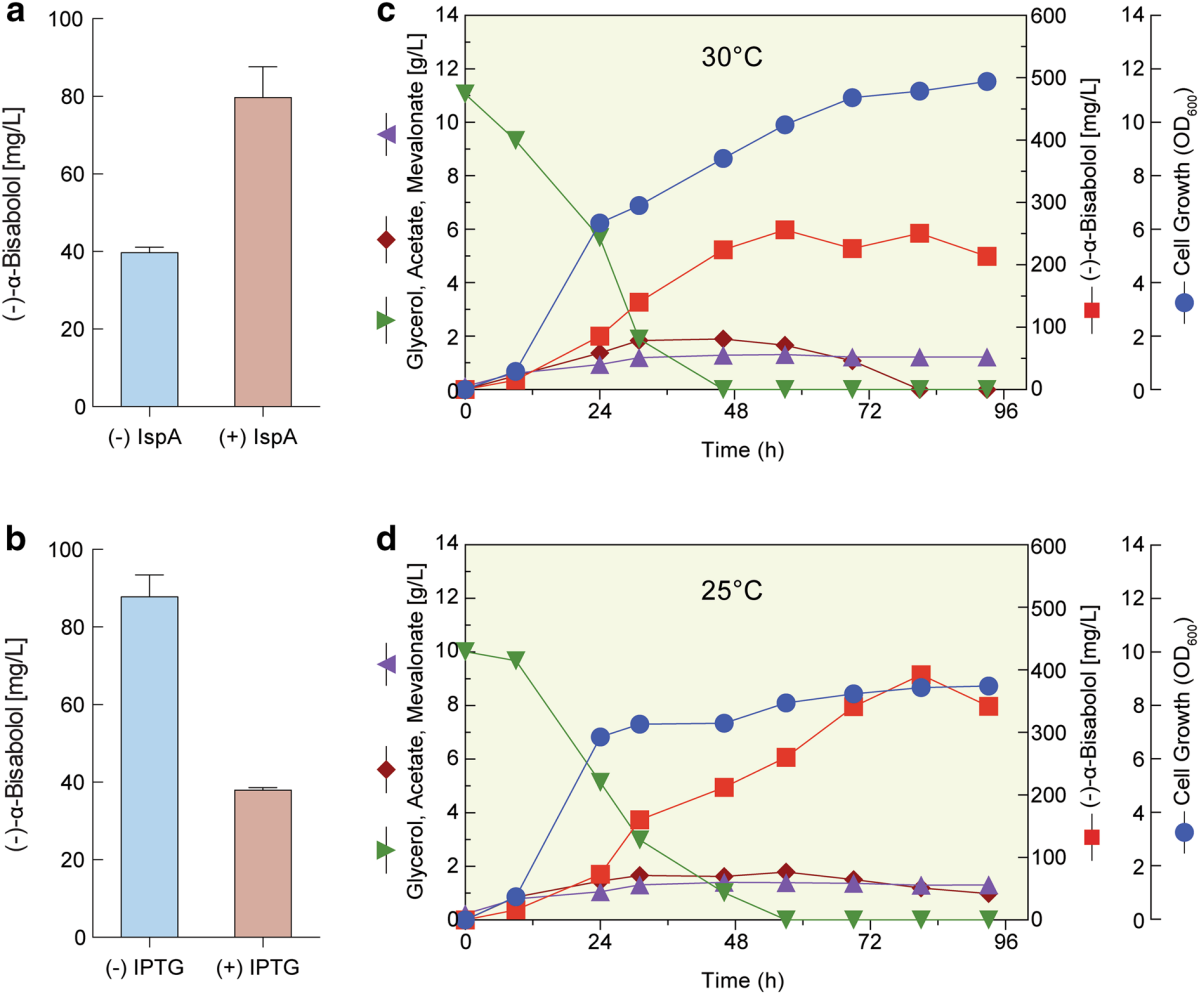
IspA overexpression and batch fermentation of engineered *E. coli*. **a** Improvement of (−)-α-bisabolol production in *E. coli* DH5α transformed with pSNA-MrBBS-IspA compared with DH5α harboring the pSNA-MrBBS plasmid. Cells were cultivated in TB medium containing 10 g/L glycerol at 30 °C for 48 h using two-phase culture without the addition of IPTG. Data represent averages from three replicate cultures; *error bars* show SD, **b** effects of IPTG addition on (−)-α-bisabolol production in *E. coli* DH5α harboring pSNA-MrBBS-IspA. Culture conditions were the same as in Fig. 6a, except for the addition of IPTG. IPTG was added to each culture to a final concentration of 0.05 mM when the OD_600_ reached 0.5. Data represent averages from three replicate cultures; *error bars* show SD, **c** time profile of batch fermentation of *E. coli* DH5α with pSNA-MrBBS-IspA in TB medium containing 10 g/L of glycerol as carbon source at 30 °C, *n*-dodecane was overlaid up to 20% (v/v) of culture volume to continuously extract (−)-α-bisabolol from the culture broth during fermentation. **d** Time profile of batch fermentation of *E. coli* DH5α with pSNA-MrBBS-IspA in TB medium containing 10 g/L of glycerol as carbon source at 25 °C. *n*-Dodecane was overlaid up to 20% (v/v) of culture volume to continuously extract (−)-α-bisabolol from the culture broth during fermentation

(−)-α-Bisabolol is produced from depyrophosphorylation of the prenyl diphosphate precursor FPP. Therefore, synthesis of FPP from IPP and DMAPP by FPP synthase, encoded by the *ispA* gene, plays a key role in the improved production of (−)-α-bisabolol. Furthermore, high concentrations of prenyl diphosphates, such as FPP, are toxic to *E. coli* cell growth [36]. FPPs needs to be efficiently converted into downstream products by TPSs to reduce its toxicity. To increase the synthesis of the cellular terpenoid biosynthesis intermediate FPP, and (−)-α-bisabolol production, overexpression of an FPP synthase, encoded by the *ispA* gene, was performed. The *ispA* gene of *E. coli* was inserted downstream of the *MrBBS* gene in the plasmid pSNA-MrBBS, resulting in the pSNA-MrBBS-IspA plasmid (Fig. 1b). This plasmid was transformed into *E. coli* DH5α and cultivated in TB medium containing 1% (w/v) glycerol at 30 °C for 48 h using a two-phase culture without the addition of IPTG. Overexpression of the *ispA* gene improved (−)-α-bisabolol production compared with pSNA-MrBBS alone. After 48 h, a concentration of 79.7 mg/L of (−)-α-bisabolol was obtained in a culture of DH5α with pSNA-MrBBS-IspA. This was twofold higher than the 39.7 mg/L of (−)-α-bisabolol produced by DH5α with pSNA-MrBBS alone (Fig. 6a), without significant difference in cell growth (Additional file 1: Figure S3D). This suggested that sufficient supply of FPP is important to improve (−)-α-bisabolol production in *E. coli*. While overexpressing the *ispA* gene, 0.05 mM IPTG at OD_600_ ~0.5 was added to induce expression of the MVA pathway and *MrBBS* genes. Similar to the results without *ispA* overexpression, (−)-α-bisabolol production decreased from 87.8 mg/L (no IPTG) to 40 mg/L (−)-α-bisabolol (0.05 mM IPTG, Fig. 6b) with no significant difference in cell growth (Additional file 1: Figure S3D). To monitor the time-course of (−)-α-bisabolol production, batch fermentation of *E. coli* DH5α harboring the pSNA-MrBBS-IspA plasmid, using a 1 L fermenter, was conducted at 30 °C in the absence of IPTG for 93 h (Fig. 6c). (−)-α-bisabolol production of 214 mg/L was achieved and cell growth reached an OD_600_ value of 11.5 after 93 h. MVA and acetate were the main byproducts, although the acetate was consumed after glycerol depletion at 48 h. The solubility of heterologous enzymes increases at lower growth temperatures because the transcription rate is slower, leading to better protein folding in *E. coli* [37, 38]. In addition, improved solubility of metabolic pathway enzymes at low temperature enhances the production yield [39]. Therefore, we performed the batch fermentation at 25 °C under the same conditions. Indeed, (−)-α-bisabolol production was 342 mg/L after fermentation for 93 h, approximately 1.6-fold higher than that obtained at 30 °C, and cell growth decreased by 25% (OD_600_ 8.7, Fig. 6d).

### Extraction and fed-batch fermentation of (−)-α-bisabolol

**Fig. 7:**
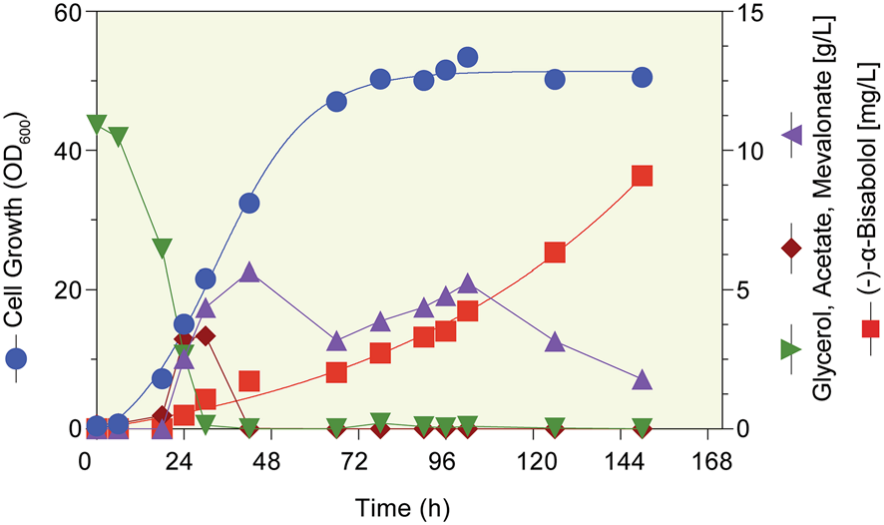
Fed-batch fermentation of *E. coli* DH5α in TB medium supplemented with 10 g/L of glycerol as initial carbon source. After complete depletion of glycerol, glycerol was fed at 3 g/L/h. Concentrations of acetate, mevalonate, and glycerol were determined by HPLC and (−)-α-bisabolol content was measured using GC. Canola oil 10% (v/v) instead of *n*-dodecane 20% (v/v) was used to overlay 30 L TB medium in a 50 L fermenter

*n-*Dodecane has been mostly used for the extraction of terpenoids produced in microorganisms due to its relatively low volatility, enabling continuous extraction over multiple days [40]. The (−)-α-bisabolol produced in flask culture and batch fermentation could be efficiently extracted from the broth to the overlaid *n-*dodecane phase. The in situ recovery of (−)-α-bisabolol from the *n-*dodecane phase during culture can reduce production costs by simplifying both the harvest and purification processes. However, *n-*dodecane is not an economically viable extracting solvent. We assessed various natural and inexpensive vegetable oils for selective (−)-α-bisabolol extraction from fermentation broth. As shown in Table 2, all tested vegetable oils showed extraction yields ranging from 96.6 to 98.8%, which are comparable to that of *n-*dodecane (99.5%). Among them, canola oil (98.8%) was the best extracting oil and was used for the extraction of (−)-α-bisabolol during fed-batch fermentation. A greener extraction of (−)-α-bisabolol plays a key role in the application of (−)-α-bisabolol produced from fermentation broth to pharmaceutical and cosmetic products. We successfully used vegetable oils as natural, cost-effective, and biodegradable extractors for in situ extraction of (−)-α-bisabolol during fermentation. Moreover, due to the difference in polarity between the fermentation broth (water based) and vegetable oils, there is a phase separation, resulting in isolation of oil from the broth containing cell debris. This in situ (−)-α-bisabolol separation from the fermentation broth can also alleviate production inhibition and improve (−)-α-bisabolol production, which will be of interest in industrial processes. To examine the production performance of (−)-α-bisabolol under conditions more relevant to industrial processes, we conducted (−)-α-bisabolol fermentation in a 50 L fermenter with a fed-batch mode. *E. coli* DH5α, containing pSNA-MrBBS-IspA, was grown at 25 °C with 10% (v/v) canola oil as an extracting solution. The final (−)-α-bisabolol titer reached 3.3 g/L after 90 h of fermentation, which was almost 8.4-fold higher than that obtained from the batch fermentation at 25 °C (Fig. 7). At the beginning of fermentation, MVA was accumulated until depletion of the initial 10 g/L glycerol. After complete depletion of initial glycerol, MVA was consumed and (−)-α-bisabolol accumulated continuously up to 9.1 g/L at the end of fermentation (Fig. 7). The specific (−)-α-bisabolol production and productivity reached 0.18 g/g dry cell weight (DCW) and 1.24 g/L/day, respectively. A previous study reported the production of 8 mg/L of (−)-α-bisabolol from *S. cerevisiae* by overexpressing the *MrBBS* gene for 4 days [15]. In this study, two-phase fed-batch fermentation of the DH5α strain expressing the *MrBBS*, entire MVA pathway, and *ispA* genes yielded 9.1 g/L of (−)-α-bisabolol after 150 h, a 1137-fold increase from that reported in the previous study. Since the (−)-α-bisabolol intermediate, mevalonate, accumulated after glycerol depletion, (−)-α-bisabolol production would increase if we increase expression of the genes of the lower MVA pathway. Such adjustments will be the content of further work to improve (−)-α-bisabolol production.

**Table 2:** Recovery yield of (−)-α-bisabolol using common vegetable oils

Extracting solution	Extraction yield (%)
Soybean oil	98.5
Canola oil	98.8
Corn oil	98.5
Sunflower oil	96.7
*n*-Dodecane	99.5

## Conclusions

In this study, we have engineered *E. coli* for *de novo* production of (−)-α-bisabolol for the first time. Introduction of the MVA pathway, (−)-α-bisabolol synthase from German chamomile, and FPP synthase turned *E. coli* into a microbial cell factory for the *de novo* production of (−)-α-bisabolol from the renewable carbon source, glycerol. Using Canola oil as an extraction solvent, the engineered *E. coli* strain produced 9.1 g/L of (−)-α-bisabolol from glycerol in a fed-batch fermentation system, and the specific (−)-α-bisabolol production and productivity reached 0.18 g/g DCW and 1.24 g/L/day, respectively. To the best of our knowledge, this is the first report demonstrating that a large amount of (−)-α-bisabolol could be produced by metabolically engineered *E. coli*. Although more work is needed to optimize the fermentation process, the strains developed in this study will serve as promising platform hosts for development of microbial production of (−)-α-bisabolol on a large scale. Moreover, a more environmentally friendly means to extract (−)-α-bisabolol, using vegetable oils, will reduce the production cost in an industrial process.

## Additional file

**Additional file 1.** **Figure S1.** Nucleotide sequence of the *E. coli* codon-optimized *MrBBS* gene. **Figure S2.** Extraction efficiency of (−)-α-bisabolol using *n*-dodecane. **Figure S3.** Cell growth during optimization of (−)-α-bisabolol production in *E. coli* DH5α expressing *MrBBS* and entire MVA pathway genes.

## Abbreviations

TPS: terpenoid synthase
IPP: isopentenyl diphosphate
DMAPP: dimethyl allyldiphosphate
MVA: mevalonate
MEP: 2-C-methyl-d-erythritol 4-phosphate
GPP: geranyl diphosphate
FPP: farnesyl diphosphate
GGPP: geranyl geranyl diphosphate
GRAS: generally regarded as safe
MrBBS: German chamomile (−)-α-bisabolol synthase gene
LB: Luria–Bertani
TB: terrific broth
IPTG: isopropyl β-d-1-thiogalactopyranoside
OD_600_: optical density at a wavelength of 600 nm
CFU: colony-forming unit
PCR: polymerase chain reaction
vvm: volume of air per volume of medium per min
GC: gas chromatography
GC–MS: GC-mass spectrometry
FID: flame ionization detector
HPLC: high performance liquid chromatography
HMG-CoA: 3-hydroxy-3-methyl-glutaryl-CoA
DCW: dry cell weight
