# Erratum to: Fermentative production and direct extraction of (−)-α-bisabolol in metabolically engineered *Escherichia coli*

**DOI:** 10.1186/s12934-017-0635-7

**Published:** 2017-01-31

**Authors:** Gui Hwan Han, Seong Keun Kim, Paul Kyung-Seok Yoon, Younghwan Kang, Byoung Su Kim, Yaoyao Fu, Bong Hyun Sung, Heung Chae Jung, Dae-Hee Lee, Seon-Won Kim, Seung-Goo Lee

**Affiliations:** 1 Synthetic Biology and Bioengineering Research Center, Korea Research Institute of Bioscience and Biotechnology (KRIBB), Daejeon, 34141 Republic of Korea; 2 Biosystems and Bioengineering Program, University of Science and Technology (UST), Daejeon, 34113 Republic of Korea; 3 Department of Chemical and Biological Engineering, Korea University, Seoul, 02841 Republic of Korea; 4 Department of Biotechnology, Chonnam National University, Yeosu, 550749 Republic of Korea; 5 Bioenergy and Biochemical Research Center, Korea Research Institute of Bioscience and Biotechnology (KRIBB), Daejeon, 34141 Republic of Korea; 6 Division of Applied Life Science (BK21 Plus), PMBBRC, Gyeongsang National University, Jinju, 52828 Republic of Korea

## Erratum to: Microb Cell Fact (2016) 15:185 DOI 10.1186/s12934-016-0588-2

Upon publication of this article [1], it was brought to our attention that Figure 7 contained an error. (−)-α-bisabolol is shown in g/L and not mg/L as incorrectly presented in the original version of the article. The correct figure is given in this erratum (Fig. 7).

**Fig. 7:**
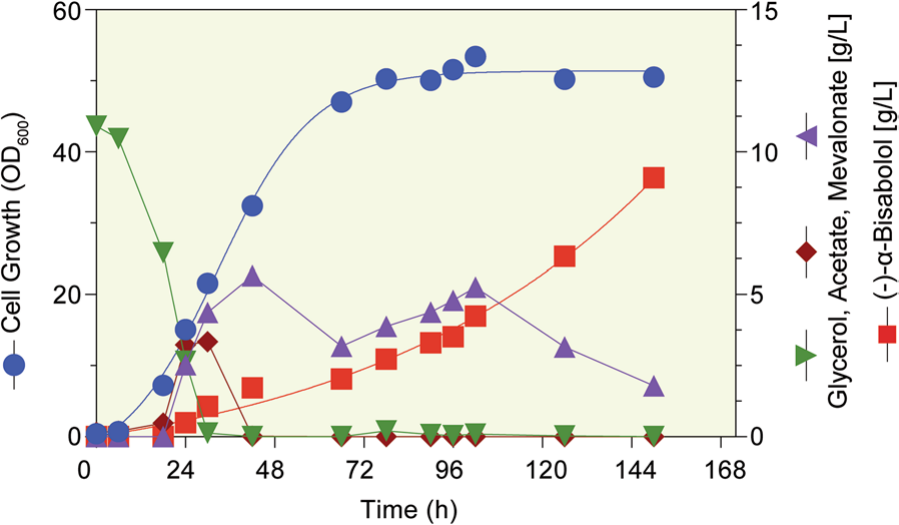
Fed-batch fermentation of *E. coli* DH5α in TB medium supplemented with 10 g/L of glycerol as initial carbon source. After complete depletion of glycerol, glycerol was fed at 3 g/L/h. Concentrations of acetate, mevalonate, and glycerol were determined by HPLC and (−)-α-bisabolol content was measured using GC. Canola oil 10% (v/v) instead of *n*-dodecane 20% (v/v) was used to overlay 30 L TB medium in a 50 L fermenter
