# Supplementary material for: Fermentative production and direct extraction of (−)-α-bisabolol in metabolically engineered Escherichia coli
Source: Microb Cell Fact. 2016 Nov 8;15:185. doi: 10.1186/s12934-016-0588-2 (PMC5101696; doi:10.1186/s12934-016-0588-2)

*Supplementary information*

**Fermentative production and direct extraction of (-)-α-bisabolol in metabolically engineered *Escherichia coli***

Gui Hwan Han^1^^†^, Seong Keun Kim^1,2†^, Paul Kyung-Seok Yoon^1,3^, Younghwan Kang^1^, Byoung Su Kim^4^, Yaoyao Fu^1^, Bong Hyun Sung^5^, Heung Chae Jung^1^, Dae-Hee Lee^1,2^*, Seon-Won Kim^6^* and Seung-Goo Lee^1,2^*

^1^Synthetic Biology and Bioengineering Research Center, Korea Research Institute of Bioscience and Biotechnology (KRIBB), Daejeon 34141, Republic of Korea

^2^Biosystems and Bioengineering Program, University of Science and Technology (UST), Daejeon 34113, Republic of Korea

^3^Department of Chemical and Biological Engineering, Korea University, Seoul 02841, Republic of Korea

^4^Department of Biotechnology, Chonnam National University, Yeosu 550749, Republic of Korea

^5^Bioenergy and Biochemical Research Center, Korea Research Institute of Bioscience and Biotechnology (KRIBB), Daejeon 34141, Republic of Korea

^6^Division of Applied Life Science (BK21 Plus), PMBBRC, Gyeongsang National University, Jinju 52828, Republic of Korea

Figure S1. Nucleotide sequence of the *E. coli* codon-optimized *MrBBS* gene.

1 ATGAGCACAC TGAGCGTCAG CACCCCGAGC TTTAGCAGCA GCCCTCTGTC GAGCGTGAAT

61 AAGAACAGCA CCAAGCAGCA TGTCACTCGT AACAGCGTGA TCTTTCACGA CTCGATTTGG

121 GGGGACCAGT TCCTGGAATA CAAAGAGAAA TTCAACGTTG CAACCGAGAA ACAGCTTATA

181 GAAGAGCTGA AAGAAGAAGT GCGTAACGAA CTGATGATTC GTGCATGTAA TGAAGCGAGC

241 CGGTATATCA AACTGATCCA GCTGATCGAT GTTGTTGAAC GTCTGGGGCT GGCCTATCAT

301 TTTGAAAAAG AGATTGAGGA AAGCCTCCAG CATATATATG TGACGTATGG TCATAAATGG

361 ACGAATTACA ACAATATTGA GAGCCTGAGT CTGTGGTTCC GCCTGCTTCG TCAAAATGGC

421 TTTAATGTTA GCTCGGATAT ATTTGAAAAT CACATTGATG AGAAAGGAAA TTTTCAGGAG

481 AGCCTGTGCA ATGATCCGCA GGGGATGCTG GCGCTGTATG AAGCGGCATA TATGCGTGTT

541 GAAGGAGAGA TCATTCTGGA CAAAGCACTC GAATTTACCA AGCTGCATCT GGGGATCATT

601 AGCAATGATC CTAGCTGTGA TAGCAGCCTA CGTACGGAAA TCAAGCAGGC ACTGAAACAG

661 CCACTGCGCC GGCGGCTGCC AAGGCTGGAA GCCGTTCGTT ACATTGCCAT TTATCAGCAG

721 AAGGCGAGCC ATAGCGAGGT TCTGCTGAAG CTGGCCAAAC TGGACTTCAA CGTTCTGCAG

781 GAAATGCACA AAGACGAATT GAGCCAAATA TGCAAATGGT GGAAAGATCT GGATATACGT

841 AACAAACTGC CCTATGTTCG TGATCGTCTG ATTGAAGGCT ATTTTTGGAT TCTGGGTATT

901 TATTTCGAAC CGCAACACTC CCGTACCCGT ATGTTCCTGA TGAAAACCTG TATGTGGCTG

961 ATCGTGCTGG ACGATACGTT TGATAATTAC GGCACCTATG AAGAGTTAGA GATCTTTACC

1021 CAAGCAGTCG AACGTTGGAG CATTACCTGT CTGGATGAAC TGCCAGAGTA TATGAAGCTG

1081 ATATATCACG AGCAATTTCG CGTGCATCAG GAAATGGAGG AAAGCCTGGA AAAGGAGGGT

1141 AAGGCCTACC AGATTCATTA TATCAAAGAA ATGGCCAAAG AAGGTACTCG TTCGCTGCTG

1201 CTGGAAGCGA AATGGCTGAA GGAAGGCTAT ATGCCTACCC TGGATGAGTA CCTGAGCAAC

1261 AGCCTGGTCA CCTGCGGCTA TGCACTGATG ACCGCACGCA GCTACGTTGC CCGTGACGAC

1321 GGCATTGTTA CCGAAGATGC ATTCAAATGG GTTGCAACGC ACCCGCCGAT TGTTAAAGCA

1381 GCATGCAAAA TTCTGCGCCT GATGGACGAC ATTGCAACCC ATAAAGAGGA ACAGGAGCGG

1441 GGACACATTG CAAGTAGCAT TGAGTGTTAC AGGAAGGAAA CCGGAGCTAG CGAAGAGGAG

1501 GCTTGCATGG ACTTTCTGAA GCAGGTTGAA GATGGTTGGA AAGTTATTAA TCAAGAAAGC

1561 CTGATGCCGA CCGATGTTCC GTTCCCTCTG CTGATTCCGG CAATTAACCT GGCACGTGTG

1621 AGCGACACCC TGTACAAAGA CAACGATGGT TATAATCATG CCGATAAAGA GGTTATAGGT

1681 TATATTAAAA GCCTGTTTGT ACATCCGATG ATAGTCTAA

Figure S2. Extraction efficiency of (-)-α-bisabolol using n-dodecane.


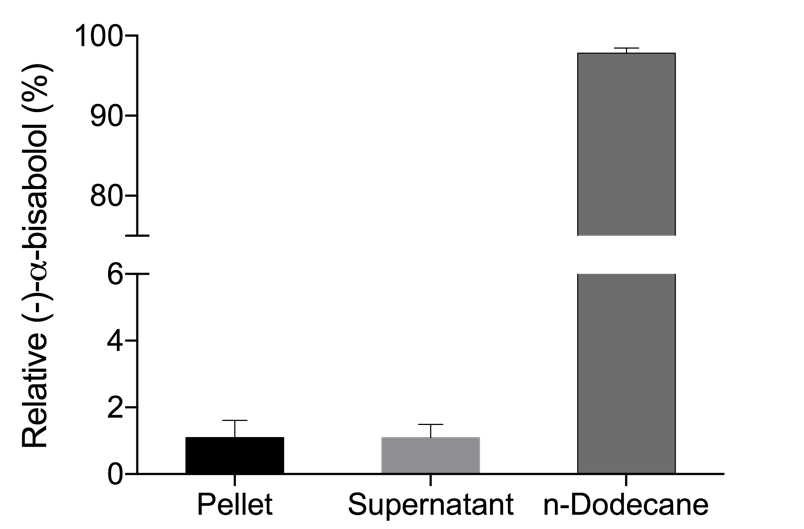


Figure S3. Cell growth during optimization of (-)-α-bisabolol production in *E. coli* DH5α expressing *MrBBS* and entire MVA pathway genes.


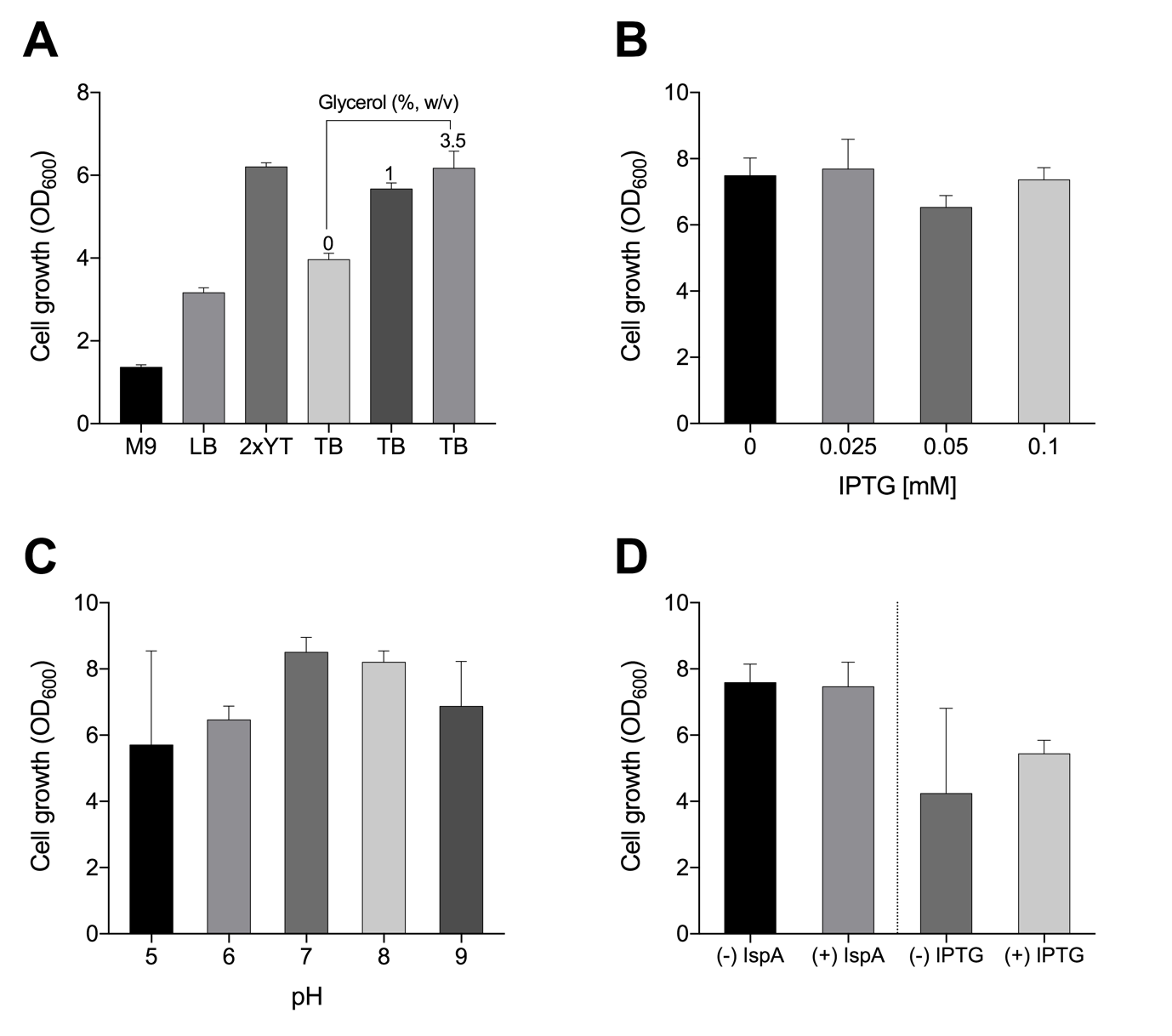

Supplement: Supplementary file 1 — Additional file 1. Figure S1. Nucleotide sequence of the E. coli codon-optimized MrBBS gene. Figure S2. Extraction efficiency of (−)-α-bisabolol using n-dodecane. Figure S3. Cell growth during optimization of (−)-α-bisabolol production in E. coli DH5α expressing MrBBS and entire MVA pathway genes. [file 12934_2016_588_MOESM1_ESM.docx]
